# A Consortium Blockchain-Based Secure and Trusted Electronic Portfolio Management Scheme

**DOI:** 10.3390/s22031271

**Published:** 2022-02-08

**Authors:** Mpyana Mwamba Merlec, Md. Mainul Islam, Youn Kyu Lee, Hoh Peter In

**Affiliations:** 1 Department of Computer Science and Engineering, Korea University, Seoul 02841, Korea; mlecjm@korea.ac.kr (M.M.M.); mainul.islam@ieee.org (M.M.I.); 2 Department of Computer Engineering, Hongik University, Seoul 04066, Korea; younkyul@hongik.ac.kr

**Keywords:** consortium blockchain, e-portfolio management system, decentralized identifier (DID), smart contract, verifiable credentials (VC)

## Abstract

In recent times, electronic portfolios (e-portfolios) are being increasingly used by students and lifelong learners as digital online multimedia résumés that showcase their skill sets and achievements. E-portfolios require secure, reliable, and privacy-preserving credential issuance and verification mechanisms to prove learning achievements. However, existing systems provide private institution-wide centralized solutions that primarily rely on trusted third parties to issue and verify credentials. Furthermore, they do not enable learners to own, control, and share their e-portfolio information across organizations, which increases the risk of forged and fraudulent credentials. Therefore, we propose a consortium blockchain-based e-portfolio management scheme that is decentralized, secure, and trustworthy. Smart contracts are leveraged to enable learners to completely own, publish, and manage their e-portfolios, and also enable potential employers to verify e-portfolio credentials and artifacts without relying on trusted third parties. Blockchain is used as an immutable distributed ledger that records all transactions and logs for tamper-proof trusted data provenance, accountability, and traceability. This system guarantees the authenticity and integrity of user credentials and e-portfolio data. Decentralized identifiers and verifiable credentials are used for user profile identification, authentication, and authorization, whereas verifiable claims are used for e-portfolio credential proof authentication and verification. We have designed and implemented a prototype of the proposed scheme using a Quorum consortium blockchain network. Based on the evaluations, our solution is feasible, secure, and privacy-preserving. It offers excellent performance.

## 1. Introduction

In recent times, electronic portfolios (e-portfolios) are increasingly used by college or university students and lifelong learners as digital online multimedia résumés that showcase their skill sets and achievements to potential employers when pursuing career opportunities. An e-portfolio [1] is a purposeful collection of digitized samples of work, demonstrations, and artifacts that highlights a person’s learning progression and achievements. It serves as evidence of students’ capabilities. For example, students can build e-portfolios that present their developed software, research papers, project reports, and multimedia (i.e., audio or video recording interviews or presentations). An e-portfolio can also be used by faculties to monitor and evaluate the effectiveness of educational programs [2,3,4]. 

### 1.1. Issues and Challenges

E-portfolios require secure, reliable, and privacy-aware credential issuance and verification mechanisms to prove learning achievements. However, with conventional e-portfolio systems, it is difficult to systematically prove educational achievements and qualifications over the Internet without relying on trusted third-party (TTP) agencies. Existing solutions [5,6,7,8] are private institution-wide centralized e-portfolio management systems (CPMSs) that primarily rely on TTPs to issue and verify credentials. Centralized TTPs are required to establish mutual trust between all participants. However, they do not enable learners to own, control, and share their e-portfolio information across organizations, which can lead to forged and fraudulent credentials. CPMSs are subject to a single point of failure (SPF), where administrators have full control and authority to modify the portfolio records without users being aware of it. They are also vulnerable because they can be targets for potential attackers, who can access the system databases by stealing user or admin credentials to tamper with the e-portfolio data. 

These limitations can be overcome using blockchain technology [9], which can enable a decentralized and immutable shared ledger where the e-portfolio data and transaction history can be recorded in a neutrally distributed and tamper-proof manner using smart contracts without any manual operations. Smart contracts [9,10] are self-executing computer programs deployed on a blockchain that runs without interference when certain predefined conditions are satisfied. Blockchain and smart contract applications can be found [9,10,11,12,13,14,15] in many fields including cryptocurrencies [11], education [12,13], identity management [13,14], and Internet of things [15]. In a blockchain-based distributed ledger, the data is timestamped and cryptographically linked in chronological blocks that are synchronized across the network. Thus, the blockchain can eliminate the need for TTPs and promote mutual trust among participant stakeholders while ensuring the reliability and decentralization of the shared ledger. In addition, digitally verifiable credentials and claims [15,16] can be leveraged to overcome the aforementioned challenges.

This study aims to address the following questions:

- How can secure, reliable, privacy-preserving e-portfolio credential issuance and verification be enabled?
- How can e-portfolio data authenticity and integrity be guaranteed?
- How can the blockchain enable learners to own, publish, and manage their e-portfolios while providing recruiters or potential employers the ability to verify the e-portfolio credentials and artifacts in a decentralized fashion?

### 1.2. Contributions

These issues are addressed by designing a decentralized, secure, reliable, and privacy-preserving e-portfolio management scheme. This study makes the following contributions:

- We propose a consortium blockchain-based decentralized, secure, and trusted e-portfolio management scheme that is integrated with recruitment platforms. This system provides users with effective methods for sharing their e-portfolios when applying to job opportunities, searching candidate profiles, matching, and recommending services for personalized user experiences.
- The e-portfolio data is stored in a cryptographically secure and machine-verifiable manner in which the elliptic curve digital signature algorithm (ECDSA) is adopted to guarantee data authenticity and integrity. The blockchain is used to record all the transactions and logs for tamper-proof, trusted e-portfolio data provenance, accountability, and traceability.
- We have designed and developed smart contracts that empower learners to completely own, publish, and manage their e-portfolios while enabling recruiters or potential employers to verify e-portfolio credentials and artifacts without relying on TTPs.
- Decentralized identifiers (DIDs) and verifiable credentials (VCs) are designed and implemented for the proposed scheme following the World Wide Web Consortium (W3C) standard specifications [16,17,18]. DIDs are used for user profile identification, authentication, and authorization, whereas VCs are used for e-portfolio credential proof authentication and verification.
- A prototype of the proposed scheme is built using the Quorum blockchain for secure, confidential, quick, and privacy-protected transactions, as well as scalable performance in the consortium network. In addition, we analyzed the privacy and security of the system and provided an evaluation of its performance. The performance is evaluated in terms of computational complexity, transaction latency and throughput, block propagation latency, e-certificate signing, and generation and verification time.

The rest of the paper is organized as follows: Section 2 elaborates on the background and related work. Section 3 describes the proposed scheme. Section 4 provides the system implementation and evaluation details. Section 5 discusses the limitations and open challenges. Section 6 concludes the paper and provides orientations for future research.

## 2. Background and Related Work

This section first elaborates on the key concepts of this study that include decentralized identifiers, verifiable credentials, presentations, and claims. Then, it elaborates on the consortium blockchain.

### 2.1. Electronic Portfolio Concepts

**Figure 1:**
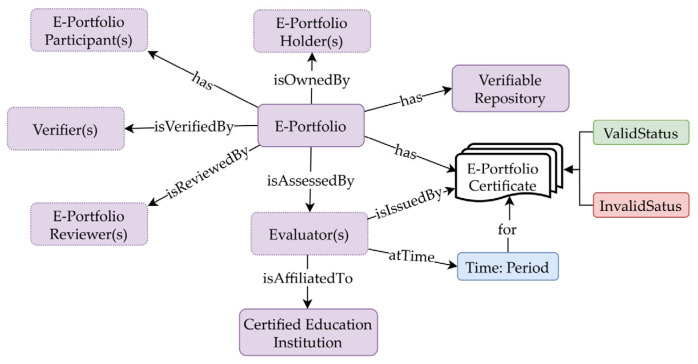
Definition of e-portfolio concepts using ontology representation.

Figure 1 presents a definition of the e-portfolio concepts adopted in this study, which is represented using an ontology model. An e-portfolio is completely owned by its holder, and it may have several contributing participants and a verifiable repository that stores relevant artifacts. It is reviewed by qualified experts and is evaluated by an accredited evaluator affiliated with a certified educational institution. The evaluated e-portfolios have associated electronic certificates (known as e-certificates) issued by evaluators; these certificates have a validity period across which they can be used to verify the e-portfolio claims.

### 2.2. Verifiable Claims 

A verifiable claim [16,17] refers to an achievement, qualification, statement, or piece of information regarding an entity that can be verified. It includes an identity, university degree, or learning achievement. A verified claim is a statement issued by a third party stating that the claim is true. Typically, a claim describes the properties (i.e., name, quantity and/or quality, and other characteristics) of an entity that establish its existence uniqueness. An entity (i.e., individual, organization, agent, or device) can make many kinds of claims. For example, a student can claim an academic degree that proves their capabilities, and an organization can claim access to verify educational records during the process of making decisions about job applications.

### 2.3. Decentralized Identifiers (DIDs)

**Figure 2:**
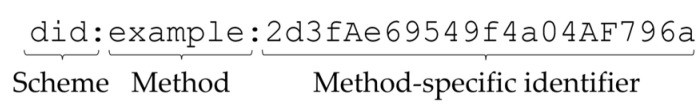
Example of a DID format.

DIDs are globally verifiable decentralized digital identities that refer to any subject, such as a person, organization, thing, or data model [18]. They can be used by individuals, organizations, and things as globally unique and trusted identifiers in many contexts, such as identification numbers (i.e., college degrees, driver’s licenses, or passports). DIDs enable entities to control their identity data and authenticate ownership by verifying cryptographic proof such as digital signatures. A DID is composed of three components: (1) the *DID URI scheme*, (2) *DID method*, and (3) *DID method-specific identifiers*, as shown in Figure 2. The URI scheme indicates the uniform resource identifier scheme, whereas the DID method defines its implementation specifications. 

An illustration of a DID document for a user profile is provided in Listing 1. It is encoded in the JSON-LD format. It is uniquely identifiable by an “id”:”did:example:2d3fAe6…f3a0c9f4a04AF796a” (line 2). The DID method type, issuer identifier, and issuance timestamp are defined in lines 3–5. Lines 6–9 specify the public key with its identifier, verification type, and key-value in multiple bases. Claims regarding the DID holder are defined in lines 10–20, and the authentication method definition in lines 21–23 includes the method type, public key, and signature value. Finally, in lines 25–30, the proof method is specified by determining the verification type, creation timestamp, creator, verification method, and the value of the signature used to sign the DID document.

**Listing 1.** Example of a DID document schema in JSON-LD ^1^ format.1: {“@context”:”https://www.w3.org/ns/did/v1”,2:   “id”:”did:example:2d3fAe6954e1Dg3f3a0c9f4a04AF796a”,3:   “type”: [“UserProfileIdentifier”],4:   “issuer”:”did:example:6384c92b19ed2Ab67f9c1V423F9e3421”,5:   “issuanceDate”:”2022-01-03T14:15:26Z”,6:   “publicKey”: [{7:     “id”:”did:example:2d3fAe6954e1Dg3f3a0c9f4a04AF796a/#keys-1”,8:     “type”:”Ed25519VerificationKey2020”,9:     “publicKeyMultibase”:”5hcZuqdn7qbXgfpEmq…wcJgR19YA3VWgB”}],10:   “claim”: {11:     “id”:”did:example:2d3fAe6954e1Dg3f3a0c9f4a04AF796a”,12:     “fullName”: “Mpyana Merlec”,13:     “email”:”abc@example.com”,14:     “profileURL”: [“https://myprofile.org”, “https://linkedin.com/myprofile”],15:     “affiliation”: {16:        “position”: “Graduate student”,17:        “institution”: {18:        “name”:”Korea University”,19:        “department”: “Computer Science and Engineering” } 20:     } 21:   },22:   “authentication”:[{23:     “type”:”Ed25519VerificationKey2020”,24:     “publicKey”:”5hcZuqdn7qbXgfpExP…aTPYTwcJgR19YA3VWgB”,25:     “signatureValue”:”4xRJc5oMwKiVXZAeUTk…JFuC9hy5cDpJeujmbaZpYDF” }],26:   “proof”:{27:     “type”:”Ed25519Signature2020”,28:     “created”:”2022-01-03T15:12:19Z”,29:     “creator”:”did:example:2d3fAe6954e1Dg3f3a0c9f4a04AF796a”,30:     “verificationMethod”:”did:example:2d3fAe6954e1…a04AF796a/issuer#01”,31:     “signatureValue”:”2G3YnyHsvgKBtm8Q2m…i3j1tER4R4aC67PckWpWx5N”}32: }^1^ JavaScript object notation for linked data—https://json-ld.org/ (accessed on 31 January 2022).

### 2.4. Verifiable Credentials and Presentations

Verifiable credentials are tamper-evident credentials whose authorship can be cryptographically verified [16,17]. They can be used to produce verifiable presentations (VPs), which are tamper-proof presentations of cryptographically verified and trusted data authorship. The verification method (VM) refers to a set of parameters that can be independently used to verify proof. Typically, VMs verify whether a verifiable credential or presentation is an authentic and timely statement of the issuer or presenter, respectively. This verification includes checking that the credential (or presentation) conforms to the specification and that the proof method is satisfied. If these conditions hold, the status check succeeds. Public-key cryptography can serve as a verification method with a corresponding digital signature. This signature instance is used to verify that the signer holds the associated private key. Listing 2 illustrates an e-portfolio VC schema encoded in JSON-LD format. This VC is uniquely identified by a DID, which is defined on line 2. On lines 3–6, the type, issuer, issuance timestamp, and expiration date of the VC are specified. On lines 7–16, the claim statements are defined by specifying the e-portfolio’s id, type, title, URL, creation timestamp, creator, reviewer(s), evaluator, and evaluation score. Finally, on lines 17–23, the proof verification method is specified by its type, creation timestamp, creator, proof purpose, verification method, and value of the signature.

**Listing 2.** Example of a verifiable credential schema in JSON-LD format.1: {“@context”:” https://www.w3id.org/credentials/v1”,2:   “id”:”did:example:16ac244ea930bc2cbf225c3d15b44674”,3:   “type”: [“ePortfolioVerifiableCredential”],4:   “issuer”:”did:example:8d6fbg6410e1071f2a0c9f4a04eE726a”,5:   “issuanceDate”:”2022-01-04T16:15:24Z”,6:   “expiringDate”:”2023-01-03T16:15:24Z”,7:   “claim”: {8:     “id”:”did:example: d6259eEA53eRi1ac8b16807fade3c70660b”,9:     “type”:”ePortfolioVerifiableCredential”,10:     “title”:”Blockchain-based e-Portfolio Management System”,11:     “portfolioURL”:”http://github.com/myportfio/ePortfolio”,12:     “created”:”2022-01-03T14:10:12Z”,13:     “creator”: [{“id”:”did:example:2d3fAe6954e1Dg3f3a0c9f4a04AF796a’”}],14:     “reviewer”: [{“id”:”did:example:1531b2D926ac6379aG4fw6520bd7a30b”}],15:     “evaluator”: [{“id”:”did:example:6384c92b19ed2Ab67f9c1V423F9e3421”}],16:    “score”:”95”},17:   “proof”: {18:     “type”:”Ed25519Signature2020”,19:    “created”:”2022-01-05T14:12:19Z”,20:     “creator”:”did:example:6384c92b19ed2Ab67f9c1V423F9e3421”,21:     “proofPurpose”:”ePortfolioCredentialVerification”,22:     “verificationMethod”:”ttps://example.edu/issuers/keys/#1”,23:     “signitureValue”:”sITJX1CxPCT8yAVPAYuN...zVBAh4vGHSrQyHUdBBPM” }24: }

### 2.5. Cryptography Primitives

To create publicly verifiable DIDs and e-certificates, we adopted Ed25519, which is a high-speed and highly secure digital signature scheme [19]. This scheme uses the Ed- wards25519, a twisted Edwards curve for signature generation and verification [20]. The twisted Edwards curve over a prime field, *F_p_* [21] is expressed in Equation (1) below:

*ax*^2^ + *y*^2^ = 1 + *dx*^2^*y*^2^ (1)

where *a*, *b* ∈ *F_p_*\{0, 1} with a ≠ d. When *a* = 1, the curve is known as an Edwards curve (untwisted). Considering *a* = −1, the curve [21] is expressed as follows:

−*x*^2^ + *y*^2^ = 1 + *dx*^2^*y*^2^ (2)

When *d* = −121,665*/*121,666 and *p* = 2^255^ −19, the curve is known as Edwards25519, which is the Edwards form of the elliptic curve Curve25519 [22]. A public key, *P_k_*(*x, y*), is generated by multiplying a base or generator point, *G*, by a secret key, *S_k_*. This is referred to as elliptic curve point multiplication (ECPM) [21], which can be defined as follows:

*P_k_ = S_k_G* (3)

where *P_k_* is also a point on the curve. It can be computed by adding *G* to itself *S_k_ −* 1 times as follows [21]:

(4) $$P_{k\;}=G+\underset{S_{k}\;-\;1\;times}{\underbrace{G+\ldots +G}}\;\;$$

If *S_k_* is expressible as a power of two, *P_k_* can be obtained by doubling *G* on itself ${log}_{2}S_{k}$ times as follows [23]:

(5) $$P_{k\;}=\underset{{log}_{2}S_{k}\;times}{\underbrace{\;\ldots 2(2(2(G)))}}\;$$

Algorithm 1 demonstrates the Ed25519 key setup and signature generation process using a secret key, *S_k_*, and message, *M* (i.e., credential or certificate) as inputs. The output of this algorithm is a two-part digital signature. The signature verification mechanism is described using Algorithm 2, in which a message, *M*, public key, *P_k_*, and signature, *S*, are provided as inputs. The verification result determines whether the given signature is valid or invalid. If the result is true, the message is signed with the exact private key that corresponds to *P_k_* and the signature is valid. If the result is false, the signature is invalid because the message is generated with a false private key, which does not correspond to *P_k_*.
**Algorithm 1.** Ed25519 key setup and signature generation [20]Curve parameter: *G*(*x, y*), *a, d, p*, order *n***Input**: secret key *S_k_*, message *M***Output**: signature *S*1: Hash *k*: *h* = *SHA*512(*S_k_*)2: *a* = *h*[0 : 32]3: *a* = *h*[32 : 64]4: *c* = *SHA*512(*b* ∥ *M*)5: Interpret *a* and *c* as integers in little-endian notation. 6: Compute public key: *P_k_* = *S_k_G*7: Generate the first part of signature: *R* = *cG*8: *h′* = *SHA*512(*R* ∥ *P_k_* ∥ *M*)9: Interpret *h′* as an integer in little-endian notation.10: Generate the second part of signature: *s* = (*c* + *ah*′) mod *n*
11: Combine signature pair: *S* = *encode*(*R*) + *encode*(*s*) 12: **return** signature *S*.

**Algorithm 2.** Ed25519 signature verification [20]Curve parameter: *G*(*x, y*), *a, d, p*, order *n***Input**: message *M*, public key *P_k_*, signature *S***Output**: True/False1: Separate signature pair: *R*, *s* = *S*[0 : 32], *decode*(*S*[32 : 64])2: *h* = *SHA*512(*R* ∥ *P_k_* ∥ *M*)3: Interpret *h* as an integer in little-endian notation.4: **return** sG=R+hQ


### 2.6. Consortium Blockchain

There are two primary types of blockchains, namely *permissioned* and *permissionless blockchains*. The former requires prior permission, whereas the latter allows anyone to download the software, join, and operate in the network. A comparative evaluation of five major blockchain platforms [16,24,25,26,27] is provided in Table 1. Considering the governance approach, we find three types of blockchain networks: *public*, *private*, and *consortium blockchain* networks. Public blockchain networks are open to the public and allow everyone with a copy of the ledger to participate as a node in the decision-making process, whereas private blockchain networks are only open to a group of individuals or participants within an organization. Consortium blockchains (a.k.a. *federated blockchains*) are hybrid blockchain networks that combine public and private blockchain network features. They are permissioned blockchain networks governed by a group of organizations that have decided to share a ledger among themselves for transactions. However, the topology, roles, and permissions of the participant nodes depend on the network requirements and the blockchain platform-supported protocols. The consortium blockchain has the following advantages [28]:

- *Access control permission*: Permissions at the network and system level are required for nodes and users to join and operate in the consortium blockchain network. A set of roles and various permissions are assigned to each user account and node.
- *Consensus-driven decentralized governance*: The blockchain is governed by the consensus of a set of authorized participating nodes in a decentralized manner. It is easy to manage and enforce the infrastructure’s rules and policies.
- *Low energy and computing resource consumption*: The consortium blockchain does not use PoW-like consensus protocols, which consume considerable energy and computing resources when solving complex mathematical puzzles.
- *Confidentiality and privacy*: Consortium blockchains provide support for transaction confidentiality and privacy. They are essential for enterprise blockchain decentralized applications (DApps).
- *High transaction throughput*: Consortium blockchain networks providing high transaction throughput as a consensus on the state of the ledger can be rapidly reached via a set of authorized validator nodes.
- *Security and scalability*: The consortium blockchain provides fault tolerance capability and better protection against disturbances even when nodes behave arbitrarily or maliciously.

In this study, the consortium blockchain is adopted to meet the requirements of our e-portfolio management scheme. These requirements include secure and confidential interactions and data exchange among several participants from several organizations that may not fully trust each other.

### 2.7. Related Work

Table 2 provides a comparison of our proposed scheme with previous studies in the literature. The design of an e-portfolio system for cooperative educational record management using a centralized cloud-based blockchain approach is presented in [29]. Arenas et al. [30] described how permissioned blockchains could be applied to centralized academic credentials issuance and decentralized verification by interested third-party organizations. Though private blockchain-based systems provide private institution-wide solutions that seem to be centralized in some ways, they suffer from SPF issues. Few studies provide decentralized e-portfolio management solutions. Chen and Zhu [31] described an approach for building a decentralized, immutable, secure personal archive management system using a blockchain. Other works [13,14,32] discussed the potential benefits of using blockchains, self-sovereign identity, and digital credentials in the education sector, where educational certification credentials are completely owned and managed by students without requiring any TTPs. However, there still exist several technical challenges that must be overcome to successfully implement these schemes. A design of a blockchain-based e-portfolio evaluation system that assesses the education and teaching processes is proposed in [33]. Other studies [34,35,36] presented practical implementations of blockchain-enabled solutions for managing life-long learning achievement records beyond transcripts and certificates. In these systems, learning activities are stored in a blockchain and access rights are managed using smart contracts. Nevertheless, these papers’ primary purpose was to investigate how a blockchain of learning logs could be shared across institutions. Furthermore, the identity and certificate issuance schemes in [36] are strictly hierarchical, and they rely on a single accreditation authority that is subject to SPF because of its centralized structure. Thus, if the accreditation authority’s private key is leaked or compromised, the entire system will be affected.

Recently, [37] proposed a blockchain-based, multilateral personal portfolio authentication scheme that guarantees the reliability, integrity, and transparency of schooling history data. Alexander et al. in [38] investigated how blockchain-enabled smart badges could help learners advance their careers by providing them with personalized recommendation services based on their learning achievements. Blockcerts, a blockchain-based solution for academic credential issuance and verification was introduced in [39]. However, it focuses on eliminating the cost of the degree verification process and did not initially consider the degree issuing institutes. Only the degree certificates issued in digital form were considered, and support for previously issued degree certificates for graduated students was not provided. Docschain [40] tackled the limitations of blockcerts by incorporating the existing degree issuance workflow with features that handle digitized copies of degree documents in optical character recognition (OCR) format. Cerberus [41] was proposed as a blockchain-based accreditation and degree verification solution. It aimed to mitigate credential fraud cases using on-chain smart contracts for credential revocation. However, most previous studies [39,40,41] lack implementation and performance evaluation details. Certain studies [42,43,44,45] introduced authentication, revision, and revocation mechanisms for academic certificate credentials stored on the blockchain to reduce fraud and improve verification efficiency. Nevertheless, most of these solutions do not provide any privacy-preserving e-portfolio management model. As they are built on public blockchain platforms, they have difficulty in ensuring user identity management, confidentiality, and privacy. To reduce the risk of compromising the credentials data, a blockchain-based scheme for self-sovereign identity (SSI) credential sharing with selective disclosure was introduced in [46], which focused on privacy.

**Table 2:** Comparison of the proposed scheme with existing works in the literature.

Paper	User-Centric	E-Portfolio	RPI ^1^	DIDs/VCs & VPs ^2^	Verifiable Repository	Smart Contract	Blockchain Network/ Platform	Privacy/Security	Implementation	Performance Evaluation
[5,6,7,8]	√	√	X	X/X	X	X	X/X	X/X	X	X
[14]	√	X	X	√/√	X	X	Public/-	√/√	√	X
[29]	√	√	√	X/X	X	X	-	X/X	X	X
[30]	√	X	X	X/X	X	X	Private/-	X/X	√	X
[31]	√	√	X	X/X	X	√	Consortium/-	-	X	X
[33]	√	√	X	X/X	X	√	Consortium/Hyperledger Fabric	√/√	Prototype	√
[34]	√	√	X	X/X	X	√	Consortium/Hyperledger Fabric	√/√	Prototype	X
[35]	√	X	X	X/X	X	√	Private/Ethereum	√/√	Prototype	√
[36]	√	√	X	X/X	X	√	Public/Ethereum	X/√	Prototype	X
[37]	√	√	X	X/X	X	√	Consortium/Hyperledger Fabric	√/√	Prototype	√
[38]	√	X	X	X/X	X	√	Public/Ethereum	X/√	Prototype	X
[39]	√	X	X	√/√	X	√	Public/Ethereum	X/√	√	X
[40]	√	X	X	X/X	X	-	Semiprivate/-	√/√	√	X
[41]	√	X	X	X/X	X	√	Private/Ethereum	√/√	Prototype	X
[42]	√	X	X	X/X	X	√	Consortium/Hyperledger Fabric	√/√	Prototype	√
[45]	√	X	X	X/X	X	√	Public/Ethereum	X/√	Prototype	√
This work	√	√	√	√/√	√	√	Consortium/Quorum	√/√	Prototype	√

^1^ Recruitment platform integration; ^2^ decentralized identifiers/verifiable credentials and verifiable presentations.

## 3. Proposed Scheme

This section elaborates on the system design of the proposed scheme, which includes the key stakeholder and role identification, system architecture, cryptography primitives, and system operations.

### 3.1. Key Stakeholder and Role Identification

The following are the key stakeholders of the proposed scheme and their legitimate user roles:

- *Accreditation authorities* certify educational institutions and evaluators by issuing verifiable credentials to them. Accreditation authorities include governments, higher education ministries, and national or international education accreditation agencies.
- *Certified educational institutions* (i.e., colleges, universities, or training centers) provide learning programs, assess learners, and certify learning results and artifacts by issuing cryptographically secure and machine-verifiable e-certificate credentials.
- *Holders* (i.e., students or lifelong learners) completely own, publish, and manage e-portfolios. Portfolio holders possess verifiable credentials, from which verifiable presentations are generated and shared with verifiers to prove ownership.
- *Evaluators* (i.e., accredited professors, instructors, or teachers) assess submitted e-portfolio claims and issue and transmit verifiable certificate credentials to the e-portfolio holders. Submitted portfolios can also be peer-reviewed by experts who are seen as *certified reviewers*.
- *Verifiers,* by receiving verifiable credentials using smart contracts, verify the e-portfolio certificate credential’s authenticity and integrity. Examples of verifiers include company recruiters, employers, and higher education supervisors.
- *Recruitment platforms* (i.e., LinkedIn, Indeed, and SaramIn) provide job or internship opportunity postings, candidate profiles searching, matching, and recommendation services.
- *A verifiable e-portfolio repository* is a system that allows users to store, share, and access e-portfolio resources. Verifiable e-portfolio repositories contain publicly, selectively, or privately published verifiable e-portfolio artifacts such as research papers, interview audio or videos, and application source codes. These repositories may require the use of DIDs and verifiable credentials. Examples of verifiable e-portfolio repositories include decentralized databases or distributed ledger-based registries.

**Figure 3:**
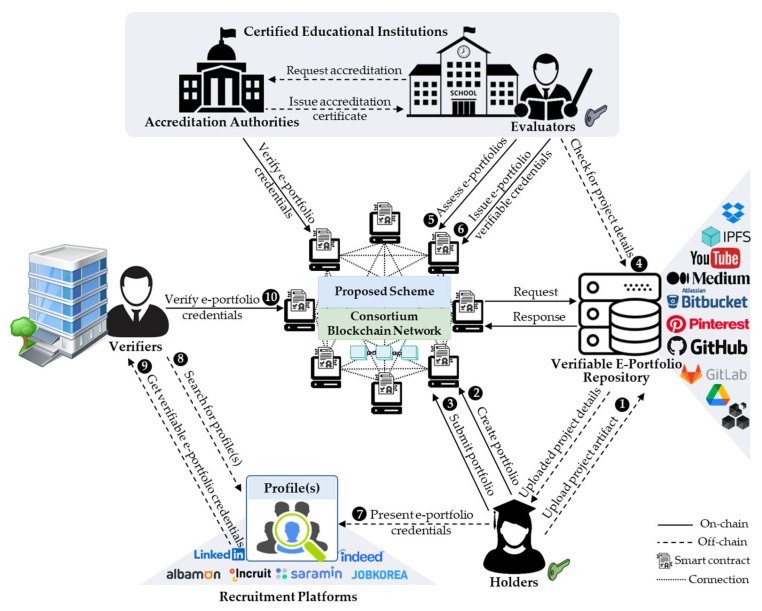
Overview of the proposed consortium blockchain-based secure and trusted e-portfolio management scheme.

Before accessing and operating the system, every user must sign up for a membership user profile with an authorized user role(s) assigned. A DID is generated for each user profile upon registration. Figure 3 provides the workflow of the proposed scheme, which is described as follows:

(1) The portfolio holders upload their e-portfolio project artifacts to the e-portfolio repositories, which are protected by private keys for ownership and access control.
(2) Subsequently, the portfolio holders create and edit e-portfolio proposals, which can be temporarily saved until their completion.
(3) After completing, holders can submit their e-portfolio proposals to be assessed by evaluators, who are accredited instructors or professors.
(4) The evaluators first check that the artifacts in the submitted e-portfolios are published in a verifiable repository.
(5) Thereafter, evaluators can assess the submitted e-portfolios by evaluating the individual student or learner’s performance and achievement(s). If there are no complaints from learners, evaluators can sign and confirm the evaluation results, which are recorded in the blockchain.
(6) E-certificates can be issued for all assessed and confirmed e-portfolios that satisfy the requirements. These certificates are signed and sent to the corresponding holders. Every e-certificate is digitally signed using the private and public key pair of the evaluator who assessed the corresponding e-portfolio. The public key is encoded in a QR code, which is embedded in the e-certificate and is used for verifying the authenticity and integrity of the e-portfolio credentials.
(7) Thereafter, the e-portfolio holders can add or publish the e-portfolio certificates on their recruitment platform profiles, which can be used as evidence of learning achievements and presented to recruiters or potential employers.
(8) Verifiers (i.e., recruiters) can search for candidate profiles that match their job requirements.
(9) Verifiers can request e-portfolio credentials from the candidate holders for authenticity and integrity verification.
(10) Using smart contracts, verifiers confirm the authenticity and integrity of the e-portfolio credentials stored in the blockchain.

### 3.2. System Architecture

**Figure 4:**
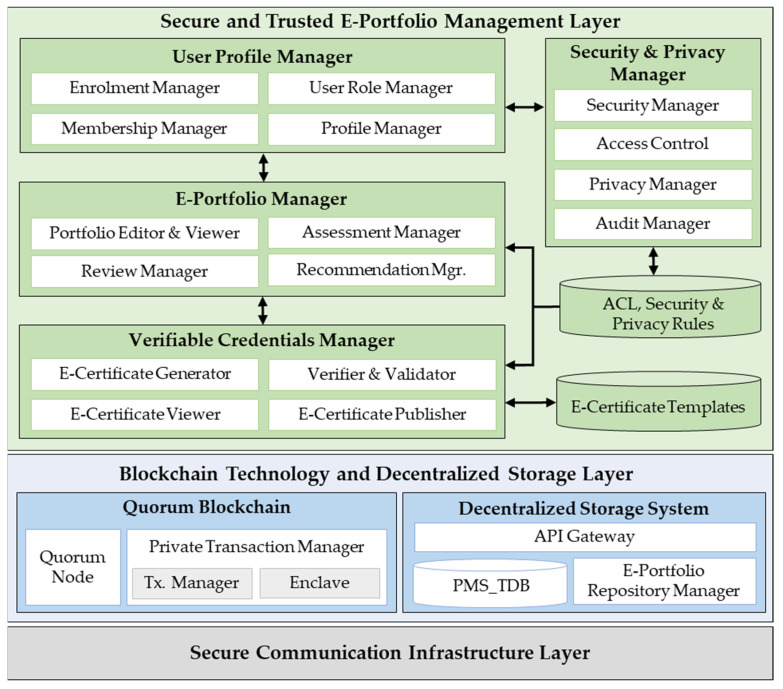
The layered system architecture of the proposed scheme.

The layered system architecture of the proposed scheme is shown in Figure 4. Aiming at modularity, the system is divided into four layers, which are described below.

1. **The secure and trusted e-portfolio management layer** provides secure and reliable e-portfolio management features, and it consists of the following modules:
  (a) The *user profile manager* is responsible for managing membership enrollments, user profiles, roles, DIDs, and credentials. It is composed of four sub-modules. (a) The *enrollment manager* provides features that support the user enrollment process; (b) the *membership manager* is used to assign and revoke membership credentials; (c) the *profile manager* manages the user profiles’ personal information, and (d) the *user role manager* is used for assigning and managing user profile roles.
  (b) The *e-portfolio manager* manages learners’ e-portfolios, which are assessed and certified by educators. It comprises the following sub-components: (a) *Portfolio editor and viewer* modules are used by learners to create and edit e-portfolios. They display the list of e-portfolios, which are filtered by category and status. (b) The *assessment manager* is used by learners and evaluators to submit e-portfolio assessment requests and to assess submitted e-portfolios, respectively. (c) The *review manager* enables reviewers to assess e-portfolios, and (d) the *recommendation manager* recommends user profiles based on their e-portfolio characteristics to provide personalized user experiences.
  (c) The *verifiable credentials manager* is responsible for generating, verifying, validating, and publishing e-portfolio certificates, and it comprises the following: (a) the *e-certificate generator* issues on-demand digital certificates for assessed e-portfolios; (b) *verifier and validator* submodules verify and validate the results of verified e-portfolio certificates; (c) the *e-certificate publisher* records e-portfolio certificates details on the blockchain; (d) the *e-certificate viewer* displays the recorded e-portfolio certificates.
  (d) The *security and privacy manager* provides custom user security and privacy management functionalities. It comprises the following modules: (a) The *security manager* provides user credentials and e-portfolio data security-related features such as authentication, authorization, confidentiality, and accountability. (b) The *access control manager* authorizes or denies access to e-portfolio data depending on the access control policies and rules embedded in the consent agreement contracts [47]. (c) The *privacy manager* helps users define and manage their privacy preferences, and (d) the *audit manager* enables users to audit their e-portfolio history in terms of when it was requested and accessed and by whom for verification. The user profile manager and security and privacy manager components extend the generic permissioned features provided by the lower layer.
2. **The blockchain technology and decentralized storage layer** provides a consortium blockchain-based immutable transaction distributed ledger and state database, which are maintained via a consensus of authorized peer nodes in the network. This layer provides an operating environment for running smart contracts.
  (a) *Quorum blockchain* [27] has two core components: (a) the *Quorum node*, which is a forked version of the *Go-Ethereum* client and is modified to support contract and transaction privacy, and (b) the *private transaction manager (PTM)*, which comprises the *transaction manager (TM)* and *enclave* sub-modules. The TM manages private transactions by allowing the access and exchange of encrypted transaction data only between authorized participant nodes. The enclave is a distributed ledger component that independently provides the cryptographic methods used for symmetric key generation, data encryption, and decryption.
  (b) The *decentralized storage system* orchestrates the verifiable e-portfolio repository data storage and access in a distributed or decentralized fashion. It comprises three core components: (a) The *API gateway* provides application programming interfaces (APIs) to access and interact with the e-portfolio repository; (b) the *e-portfolio repository manager* manages the e-portfolio repository contents, and (c) the *e-portfolio management transactional database (PMS_TDB)* is a distributed database used to store the transaction records before being committed and pushed to the blockchain ledger.
3. **The secure communication infrastructure layer** provides a secure and reliable communication service based on dedicated legacy Internet secure channels or a novel secure Internet architecture, known as SCION (scalability, control, and isolation on next-generation networks) [48], that enables private paths.

### 3.3. Working Operations

**Figure 5:**
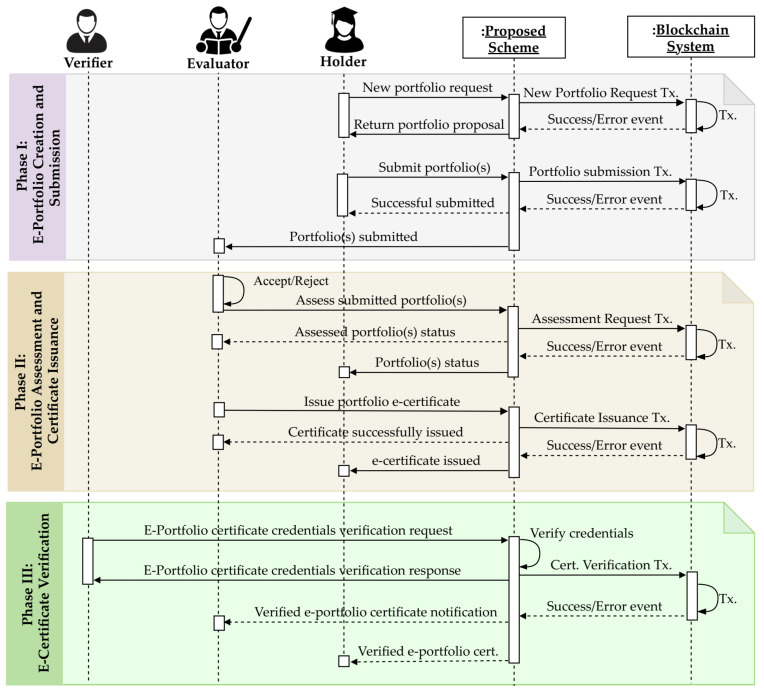
Operational working sequence diagram of the proposed scheme.

Figure 5 depicts the operational working sequence of the proposed scheme. It consists of the three phases described below.

#### 3.3.1. E-Portfolio Creation and Submission

As shown in Figure 5, to register an e-portfolio, the holder creates and submits a new portfolio request that is processed by the system, which returns an e-portfolio proposal. After editing, the holder submits the e-portfolio proposal, which is reviewed and assessed by specific reviewers and evaluators, respectively. The detailed e-portfolio registration process is provided in Algorithm 3. This algorithm receives the portfolio input parameters 〈$P_{id}{,\;P}_{N}{,\;\tau ,\;\omega ,\;\rho ,\;P}_{k},\;s,\;\delta ,\;\nu$〉 described in Table 3. Then, it checks if the portfolio ID *P_id_* does not exist in the ledger, after which the *newPortfolio* function is called to execute the transaction that stores a new portfolio instance in the blockchain. Upon successful execution of the transaction, the system emits a *newPortfolioCreated* event and returns the transaction hash value, which is stored in PMS_TDB. By contrast, in the case of transaction execution failure, an error message is returned, and the smart contract is reverted to its initial state.
**Algorithm 3.** E-portfolio registrationSmart contract parameter: $\langle C_{a}{,\;A}_{a}\rangle$**Input**: $\langle P_{id}{,\;P}_{N}{,\;\tau ,\;\omega ,\;\rho ,\;P}_{k},\;s,\;\delta ,\;\nu \rangle$**Output**: Transaction execution state 1: Collect the completed portfolios from the transactional database:  P=SELECT*FROM PMS_TDB where *pf_status*=“COMPLETED”2: **while (***P_id_, P_N_, τ, ω, ρ, P_k_, s, δ, ν***) do**3:     Check whether *P_id_* exists in the blockchain:4:     *E ←sc.getPortfolioInfo(P_id_)*5:     **if**
*E* ! = *NULL*
**then**6:     Execute the transaction *T* to store *P_id_* instance in blockchain:7:     *T ← sc.newPortfolio*(*P_id_, P_N_, τ, ω, ρ, P_k_, s, δ, ν*)8:     **if**
*err* ! = *NULL*
**then**9:       **return**
*errorMessage(err.Text)*10:     **else**11:       Emit *sc.newPortfolioCreated*(*P_id_, ω, ρ*)12:       Store *T_h_* in the PMS_TDB transactional database13:     **end if**14:     **else**15:     **return** “*P_id_* already exists” 16:     break exit()17:     **end if**18: **end while**

#### 3.3.2. E-Portfolio Assessment and Certificate Issuance

**Figure 6:**
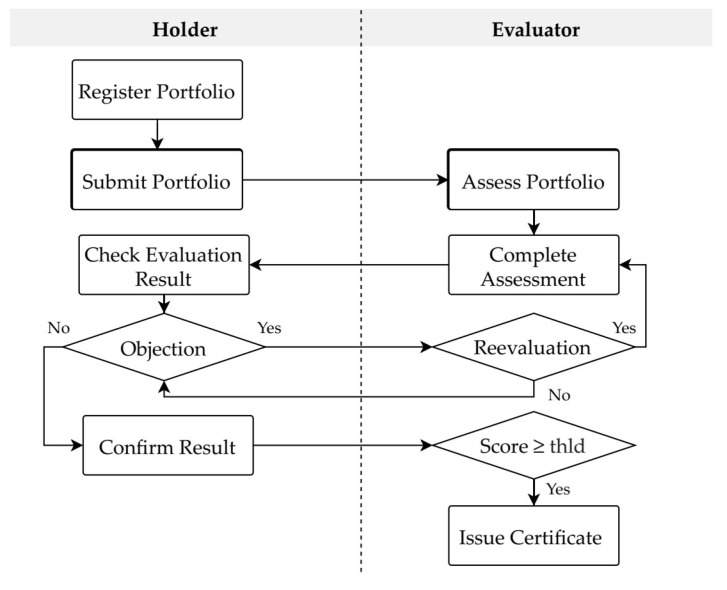
E-portfolio assessment and certificate issuance flowchart.

Evaluators can accept or reject e-portfolio assessment requests. Upon accepting an assessment request, the evaluator assesses the corresponding e-portfolio submission. Figure 6 depicts the e-portfolio assessment and certificate issuance processes of the proposed scheme. After completing the assessment, the e-portfolio evaluation result is temporarily saved in PMS_TDB, and the holder is notified for confirmation. In the case of an objection, the holder can request a reevaluation. When the evaluation result is confirmed by the holder, the evaluator can confirm and push the result to the blockchain. The e-portfolio certificate is issued only if the evaluation score is higher than or equal to a predefined threshold score value. Algorithm 4 describes the e-certificate issuance process, in which the portfolio and certificate identifiers 〈$P_{id}{,\;C}_{id}$〉 are inputs, and the output is the transaction execution state. First, the system inquires whether the certificate has already been issued for *C_id_* by calling the *getCertificateInfo* function. If no certificate exists for the given *C_id_*, then the system determines whether the corresponding e-portfolio *P_id_* has been recorded in the blockchain. If *P_id_* exists in the blockchain, the portfolio information, *P*, is collected by calling the *getPortfolioInfo* function with *P_id_*. Finally, a certificate is issued by the system executing a transaction in which *P* is passed to the *issuePortfolioCertificate* function of the smart contract.
**Algorithm 4.** E-certificate issuanceSmart contract parameter: $\langle C_{a}{,\;A}_{a}\rangle$**Input**: $\langle P_{id}{,\;C}_{id}\rangle$**Output**: Transaction execution state1: *P← sc.getCertificateInfo*(*C_id_*)2:  **if**
*P* == *NULL*
**then**3:    *P*
*← sc.getPortfolioInfo*(*P_id_*)4:    **if**
*P* == *NULL*
**then**5:     **return** “No portfolio issued for *P_id_*.”6:    **else**7:     *sc.issuePortfolioCertificate*(*P*)8:     **end if**9: **else**10:     **return** “Certificate already issued for *C_id_*.”11: **end if**

#### 3.3.3. E-Certificate Generation and Verification

The e-certificate generation process is described in Algorithm 5. A proper certificate template *C_t_* (on which the portfolio details will be embedded) is selected based on the portfolio holder’s affiliation. The portfolio information, *P*, is collected by calling the *getCertificateInfo* function with the corresponding *C_id_*. *P* is signed using the secret key, *S_k_*, of the evaluator (i.e., professor). A QR code that includes the signed *P*, evaluator’s public key, *P_k_*, and signature, *S*, is generated. Finally, the portfolio details and QR code are embedded on the template, and later, an e-certificate is created.
**Algorithm 5.** E-certificate generationSmart contract parameter: $\langle C_{a}{,\;A}_{a}\rangle$**Input**: $\langle C_{id}{,\;S}_{k}{,\;P}_{k}{,\;C}_{t}\rangle$**Output**: E-certificate1: *P*
*← sc.getCertificateInfo*(*C_id_*)2: **if** *E* ! = *NULL* **then**3:   Generate signature: *S* = *Ed25519*.*sign*(*S_k_, P*)4:   Generate QR code: *QR* = $\langle P_{k},\;P,\;S\rangle$5:     Embed *P* and *QR* on *C_t_*. 6: **else**7:     **return** “No certificate issued for *C_id_*.”8: **end if**

Algorithm 6 demonstrates the e-certificate verification process. To verify an e-certificate, a verifier first extracts the portfolio information, *P*, public key, *P_k_*, and signature, *S*, from the QR code using a QR code reader. The system checks whether *P* matches the portfolio details specified on the certificate. If *P* is the same as the portfolio details written on the certification, then *P_k_* is validated using the DID of the corresponding evaluator. If *P_k_* is genuine, *S* is verified using *P_k_*. If the verification process succeeds, the certificate is considered to be genuine; otherwise, it is rejected by the system.
**Algorithm 6.** E-certificate verification**Input**: Certificate, evaluator’s DID**Output**: Verification result 1: Read the *QR* code on the certificate.2: Extract information $\langle P_{k},\;P,\;S\rangle$ from the QR code.3: **if**
*P* matches the certificate details **then**4:   **if**
*P_k_*
∈ evaluator’s DID **then**5:       Verify the signature: *v* = *Ed*25519*.verify*(*P, P_k_, S*)6:       **if**
*v* == *T rue*
**then**7:         **return** “Certificate is genuine.”8:       **else**9:         **return** “Invalid signature”10:       **end if**11:     **else**12:       **return** “*P_k_* is not genuine.”13:   **end if**14: **else**15:   **return** “Portfolio does not match.”16: **end if**

## 4. Implementation and Evaluation

In this section, we describe the implementation details and provide the privacy and security analysis of our scheme. Finally, the system performance evaluation is provided.

### 4.1. Implementation Details

Table 4 describes the experiment environment setup used to evaluate the performance of the proposed scheme. The proof-of-concept of our scheme was built using *GoQuorum* [49], an open-source, Ethereum-based, permissioned blockchain platform with advanced enterprise-grade features that enable contract and transaction privacy, pluggable consensus protocols (i.e., *IBFT* [50] and *RAFT* [51]), and scalable performance. A consortium blockchain network infrastructure was deployed in a *Docker container* environment. This network consisted of six peer nodes and their transaction manager and ethlogger nodes. The network management, monitoring, and reporting tools are described in Table 5. Every peer node has a digital wallet containing the user profile credentials, key pairs, and associated quorum account addresses. *Tessera* [52] was adopted as a transaction manager to encrypt/decrypt and broadcast private transactions to authorized participant nodes in the consortium blockchain network using *Constellation* [53], a self-managing and peer-to-peer communication system for secure messaging. The *RAFT* [51] consensus protocol was adopted for its dynamic on-demand block creation time, fast consensus, and immediate transaction finality. *Cakeshop* [54] was used to explore, monitor, and manage all the nodes and resources in the consortium blockchain network. In addition, the Cakeshop built-in *Sandbox* integrated development environment was used to develop, compile, and deploy the smart contracts, which were coded in *Solidity* language. The JSON-RPC, Web3, and REST APIs were used by a DApp to interact with the smart contracts and blockchain ledger. DApp was developed using *Flask*, a Python framework for building web applications. A *MongoDB* server was deployed when implementing the PMS_TDB distributed database.

### 4.2. Privacy and Security Analysis 

In this subsection, we analyze various privacy and security features of our system, man-in-the-middle (MITM) attack resilience, and smart contract security.

- *Privacy preservation*: To preserve user privacy, the identity and personal information of e-portfolio holders (learners) or evaluators from their learning institutions are not shared across different organizations. Instead, we used DIDs and VCs for user profile identification, authentication, and authorization. Verifiable claims are used for e-portfolio credential proof authentication and verification. Furthermore, the proposed solution enables users to define personalized privacy and security settings, which are supported by the underlying permissioned blockchain.
- *Authentication/authorization and accountability*: To access and operate the system, each user must register for a membership user profile with an authorized user role(s) assigned. As all transaction histories are logged in the blockchain-based distributed ledger to ensure traceability, all of the participants are accountable for their activities. GoQuorum [49] provides enhanced network permission models for node and user authentication and authorization.
- *Data authenticity and integrity*: The e-portfolio information recorded on the blockchain cannot be arbitrarily modified because the blockchain is a tamper-proof distributed ledger. To guarantee user credentials and e-portfolio data authenticity and integrity, all the transaction history and logs are saved in the blockchain for its tamper-resistance, trusted data provenance insurance, and accountability features.
- *Availability and reliability*: Conventional certification systems are not publicly verifiable without TTPs, whereas the proposed scheme provides a privacy-preserving self-sovereign e-certificate issuance and verification model that is decentralized, reliable, and secure. Using smart contracts, the verifiers or recruiters can easily validate a certificate by verifying the embedded QR code without interacting with the evaluator or issuing institution.
- *MITM attack resilience*: Making a false claim using a duplicate e-certificate is possible; however, it will not be successful. It is possible to modify the embedded e-portfolio data on the certificate or change the signature. In the case of a duplicate or tampered certificate, the signature cannot be verified by the evaluator’s public key because the portfolio information is not signed with the correct evaluator’s secret key. The only way to generate an illegitimate e-certificate is to steal the secret key of the corresponding evaluator. Therefore, evaluators are advised to store their secret keys in safe devices. Storing secret keys in insecure devices or sharing the keys with others may create opportunities for unauthorized holders to claim illegitimate e-certificates.
- *Smart contract security*: The security analysis of our solidity smart contracts, which are deployed on the quorum blockchain, was performed using the latest *SmartCheck* [55] and *VeriSmart* [56] tools. Smart contracts are secure against well-known vulnerabilities such as integer overflow, integer underflow, access control, unchecked low-level calls, reentrancy attacks, and timestamp manipulation. Furthermore, as quorum eliminated the transaction fees existing in the Ethereum public blockchain, users will never run out of gas [27].

### 4.3. Performance Evaluation

The system performance is evaluated considering the following performance metrics:

#### 4.3.1. Computational Complexity Analysis

We analyzed the computational complexity of core transactions that interact with the blockchain. From Table 6, it can be observed that the complexity of an e-portfolio registration transaction is *O*(1) corresponding to a single read-write operation. It is necessary to verify whether a given *P_id_* portfolio record does not exist in the ledger and then write a new portfolio record instance in the blockchain. However, the e-certificate issuance transaction complexity is *O*(2), indicating that two read-write operations are required. One read operation collects the e-portfolio details for a given *P_id_*, and the other verifies if the given *C_id_* certificate does not already exist in the blockchain to avoid duplication. For write operations, one changes the e-portfolio status after its certificate has been issued, and the other records the information from the issued certificate to the blockchain. Finally, the e-certificate verification transaction complexity is *O*(1), which consists of a single read-write operation. This operation verifies the e-certificate authenticity and validity of a given *C_id_* and logs the verification result in the blockchain. As the transaction’s algorithm complexity affects its execution time and latency, the evaluation results reveal that the computational complexity of the proposed algorithms is linear and increases linearly with the size of the input transactions.

#### 4.3.2. E-Portfolio Transaction Latency and Throughput

**Figure 7:**
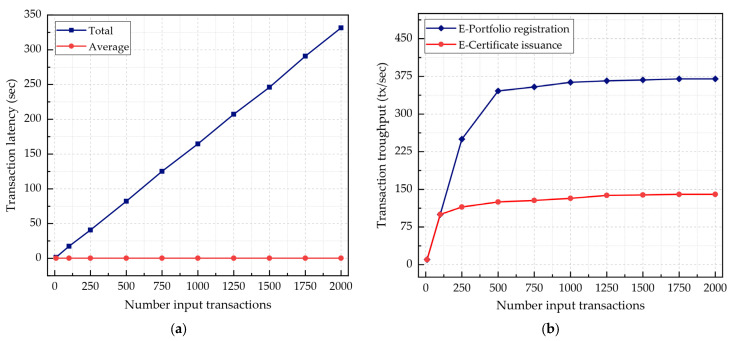
Transaction latency and throughput: (**a**) e-portfolio registration transaction latency measurements with variable input transaction rates; (**b**) e-portfolio registration and e-certificate issuance transaction throughput measurements with variable input transaction rates.

The transaction latency is the time that elapses between a transaction request submission and the response after the transaction is successfully executed, confirmed and included in a block, and committed to the blockchain [27]. By contrast, the transaction throughput is the number of transactions processed per second (TPS) by the blockchain network. In this experiment, we aimed to analyze the impact of input transaction rates on the transaction latency and throughput of the blockchain network. We generated mixed input transaction workloads (read and write) ranging from 1 to 2000 tx/s to test the system’s response to stress. The experiment was repeated three times, and then the average latency and throughput of the transactions were calculated. Table 7 provides the performance values of the e-portfolio registration transaction. In Figure 7a, we assessed the e-portfolio registration transaction latency performance under variable input transaction rates. An e-portfolio registration transaction with a size of 2.380 KB takes 164.677 ms to be processed, included within a block, and committed to the blockchain. It is found that the transaction latency scales linearly as the input transaction rate increases. Figure 7b shows the throughput measurements for the e-portfolio registration and e-certificate issuance transactions. For both transactions, the throughputs scale linearly with the low transaction input rates at approximately 132 tx/s and 363 tx/s for the e-portfolio registration and e-certificate issuance transactions, respectively. Nonetheless, the throughput does not increase considerably until a maximum of 140 tx/s and 370 tx/s for the e-portfolio registration and e-certificate issuance transactions, respectively.

#### 4.3.3. Block Propagation Latency

**Figure 8:**
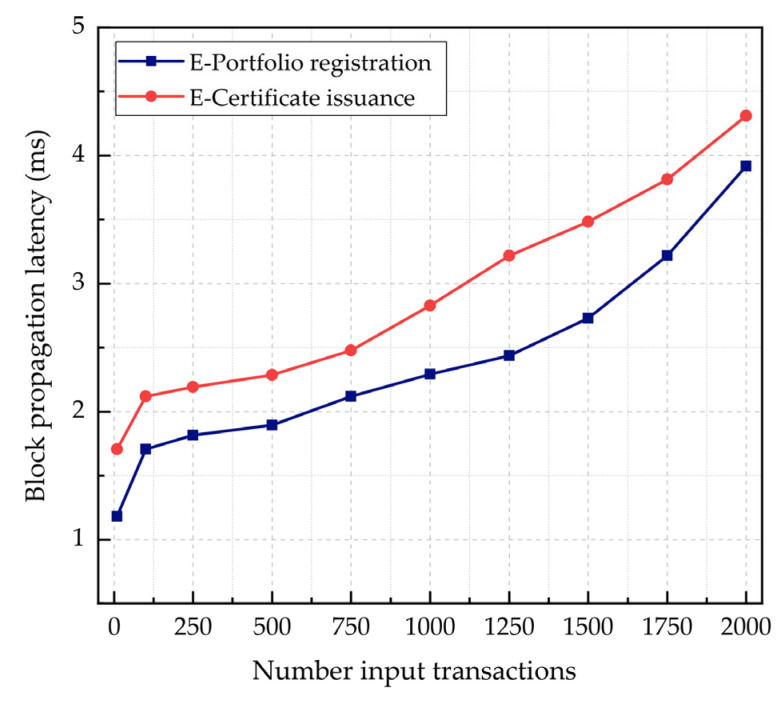
E-portfolio registration and e-certificate issuance transaction block latency measurements with variable input transaction rates.

GoQuorum [49] supports fault-tolerant consensus protocols, such as RAFT and IBFT. The block creation and validation are guaranteed, as the network can still operate and reach consensus even in the presence of adversary nodes. With RAFT, blocks are minted on-demand no more frequently than every 50 ms [51]. RAFT offers faster block times and does not create unnecessary empty blocks, whereas, with IBFT, blocks are always minted by validators at regular intervals, even in the absence of transactions on the network [50]. The IBFT block time is by default 1 s. The block propagation latency is the average time taken for a block to be produced and broadcast throughout the blockchain network [27]. As the block time parameter affects the overall latency and throughput of the system, we investigated the impact of the input transaction rates on the block propagation latency. The number of transactions per block is set by default to one for simplicity. The e-portfolio registration transaction block size is 4.251 KB, and the propagation latency is on average 1.081 ms, as indicated in Table 7. Nevertheless, the e-certificate issuance transaction-related block size is 4.768 KB with 1.705 ms of propagation latency, as given in Table 8. Figure 8 shows the block propagation latency measurements for e-portfolio registration and e-certificate issuance transactions. The block propagation latency scales as the number of input transactions increases. However, the network exhibits, on average, a 36.59% lower block propagation latency for e-portfolio registration transactions when compared to e-certificate issuance transactions.

#### 4.3.4. Certificate Signing, Generation, and Verification Time

**Figure 9:**
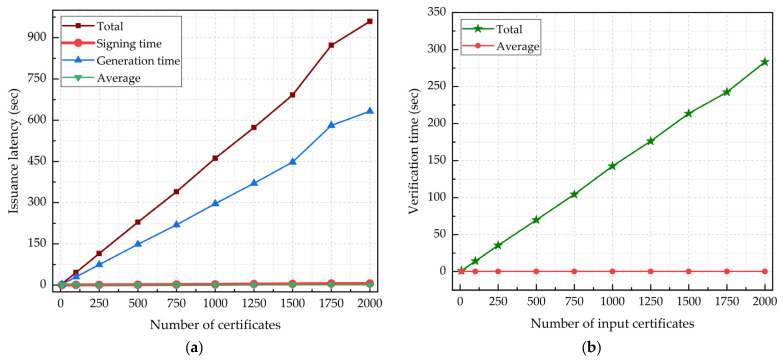
E-portfolio certificate signing, generation, and verification time: (**a**) e-certificate issuance transaction latency measurements with variable input transaction rates; (**b**) e-certificate verification time measurements with variable input certificate numbers.

Certificate issuance latency is the overall time needed to sign, generate an e-certificate document file, and record all the transaction details in the blockchain. Table 8 summarizes the e-portfolio certificate signing, generation, and verification time performances. Using the Ed25519 algorithm, it takes an average of 2.470 ms and 287.931 ms, respectively, to sign and generate a certificate, resulting in a 446.744 ms latency per certificate issued. The average transaction size is 2.896 KB, resulting in a block size of 4.768 BK, which corresponds to 1.705 ms of block latency. In Figure 9a, we analyzed the e-certificate issuance transaction latency using variable input transaction rates. The results show that the total e-certificate issuance transaction latency scales linearly as the input transaction rate increases. Furthermore, the e-certificate signing time and the generation time are 0.55% and 65.27% of the total issuance latency time, respectively. In Figure 9b, we evaluated the e-certificate verification time using a variable number of input certificate verification requests. The verification request transaction time measurements exhibit a linear progression that is proportional to the number of input certificates. The average time needed to verify the Ed25519 signature embedded in the QR code for each certificate is 136.071 ms.

## 5. Limitations and Open Challenges

Our blockchain-enabled secure and trusted e-portfolio management system could act as an anti-counterfeiting digital twin to ensure that portfolio data has not been tampered with. However, blockchain security [55,56,57,58,59,60] still imposes substantial challenges that require further research. These challenges occur at four levels:

### 5.1. Process Level

(a) *Smart contract vulnerabilities*: As smart contracts are leveraged to automate processes, they must be correctly coded and systematically verified to ensure that they can run accurately without bugs and security vulnerabilities before deployment. In addition, smart contracts are immutably stored on the blockchain after deployment and cannot be updated or upgraded to patch bugs or security vulnerabilities. Smart contract security [55,56,57,58] is a serious issue that must be considered in terms of the entire system lifecycle from requirement analysis to coding, deployment, and maintenance.
(b) *Privacy and security policies*: Users must define adequate privacy and security policies to protect their resources. This process might be challenging if the system does not provide sufficient support.
(c) *Operation standards and regulations*: Operational and regulatory standards are required for a massive adoption of blockchain technology in education for lifelong records keeping and self-sovereign credentials issuance and verification.

### 5.2. Data Level

Blockchain security [58] at the data level includes access control, key management, encryption, and consensus algorithms.

(a) *Access control and key management*: Efficient access control mechanisms are required for authentication and authorization. User-friendly cryptographic key management schemes are needed to confidentially encrypt and decrypt user data.
(b) *Blockchain oracle*: The data exchange between the off-chain and on-chain environment is enabled by smart contracts, which must be properly integrated within DApps to avoid the blockchain oracle issue [59].
(c) *Consensus algorithms*: Robust and fault-tolerant consensus mechanisms are critical for data synchronization among participating nodes and to maintain the consistency of the ledger.

### 5.3. Infrastructure Level

(a) *Standardization*: Standards are essential for enabling the resolution, authentication, and interoperability of DIDs and VCs across various domains over the Internet. Cryptographic keys are essential for creating the digital signatures used to verify user identity and prevent data tampering.
(b) *Organization, node, and account permissions*: The consortium blockchain should support enhanced permission features at the network, organization, node, account, and resource levels depending on business needs. Organizations should be able to create sub-organizations and assign roles to their nodes and accounts. Private contracts and transactions should be visible and accessible only to authorized users.
(c) *Blockchain network and communication infrastructure security*: Although the proposed scheme has leveraged smart contracts and a distributed ledger to enable decentralization, in some cases, integrated recruitment platforms and/or verifiable repository services (e.g., GitHub or Google Drive) may still be centralized; these services may be targeted by distributed denial-of-service (DDoS) attacks to render e-portfolio resources unavailable. However, this vulnerability can be mitigated using a secure and highly available Internet architecture like SCION [48], which provides secure multi-communication paths that cannot be hijacked and guarantees communication despite DDoS attacks. The blockchain security issues [60] also require further research.

### 5.4. Physical Level

As the identity and/or certificate information on a document can be forged before being recorded in the blockchain, tamper-resistant chemical signatures (in addition to the physical QR code) may be useful for physical certificate counterfeiting prevention in a distributed context, as proposed in [60].

## 6. Conclusions and Future Research

In this study, we have designed and implemented a decentralized, secure, and reliable e-portfolio management scheme powered by a consortium blockchain. Smart contracts were leveraged to enable learners to design, own, publish, and control their portfolios over a lifetime of learning and work. Furthermore, the e-portfolio credentials and artifacts are verifiable by potential employers and recruiters without relying on trusted third-party support. The immutable blockchain-enabled shared ledger records the complete transaction history to provide accountability, traceability, and tamper-resistant trusted data provenance. The reliability and decentralization promoted by the blockchain guarantee the long-term availability of e-portfolio resources for holders and investors. In addition, to preserve user privacy, DIDs are used to identify, authenticate, and authorize user profiles, whereas VCs enable e-portfolio credential proof authentication and verification. We analyzed the system privacy and security, and evaluated its performance considering the computational complexity, latency, and throughput of transactions; block propagation latency; e-certificate signing; and generation and verification time. The evaluation results demonstrate that our proposed scheme is feasible and secure, protects user privacy, and exhibits superior performance. This study is a step towards smart self-sovereign e-portfolio management as well as secure and reliable educational data exchange both nationally and internationally. However, there still remain non-technical challenges such as operation standardization, governance, and regulation. Future research directions are as follows:

- We plan to investigate recommended algorithms for providing efficient matches between learners and educators through online education platforms and between job seekers and employers through trusted skill marketplaces.
- Automated tools for auditing and fixing smart contract vulnerabilities are essential for ensuring system security at the process level.
- The design of user-friendly and efficient key management approaches would enable users to take advantage of our proposed solution.

## 7. Patent

In, H.P.; Merlec, M.M., “System for portfolio management based on blockchain and blockchain-based portfolio management system integrated with recruitment platforms.” Korean Patent, No. 10-2021-0140310, 20 October 2021.

## Figures and Tables

**Table 1:** Comparative evaluation of five major blockchain platforms.

Features	Ethereum	Hyperledger Fabric	Hyperledger Indy	Corda	Quorum
Industry	Cross-industry	Cross-industry	Digital identities (DIDs)	Financial	Cross-industry
Mode of operation (ledger)	Permissionless (public)	Permissioned (private)	Permissioned (Public)	Permissioned (private)	Permissioned (public/private)
Consortium network support	X	√	N/A	√	√
Decentralization	Decentralized	Partially	Decentralized	Partially	Decentralized
Consensus protocols	PoW ^1^	Pluggable	PBFT ^4^	Notary-based	Pluggable
Transaction throughput (TPS)	~20 tps	>2000 tps	-	~170 tps	~1000 tps
Smart contract support	√	√	X	√	√
Transaction/smart contract privacy	X/X	√/√	√/X	√/√	√/√
Native cryptocurrency	ETH ^2^	N/A ^3^	N/A	N/A	ETH

^1^ PoW: proof-of-work; ^2^ ETH: Ethereum cryptocurrency; ^3^ N/A: not available; ^4^ PBFT: practical Byzantine fault tolerance.

**Table 3:** Notation description.

Symbol	Description
*Ca* *, Aa, SC*	Smart contract address, account address, smart contract
*P_id_,P_N__,_* *δ*	E-portfolio identifier, e-portfolio title name, e-portfolio status
*C_id_* *,* *C_t_*	E-certificate identifier, e-certificate template
*τ*	Registration timestamp (date and time)
*ω*	E-portfolio creator user profile identifier
*ρ*	Evaluator’s (i.e., professor or instructor) name
*P_k_* *_,_* *S_k_* *, S*	Public key, secret or private key, digital signature
*s* *, ν*	Evaluation score, e-portfolio access URL (uniform resource locator)
*T_h_* *,* *QR*	Transaction hash, quick response code
*PMS_T DB*	E-portfolio management system transactional database

**Table 4:** Experiment environment setup.

Hardware	Description
CPU/ GPU	AMD^®^ Ryzen 7 1700-8 Core/NV132
RAM/SSD	64 GB/2 TB
Network interface	I211 Gigabit Network
**Software**	**Description**
OS	Ubuntu 20.04.3 LTS, 64bit
Number of nodes	6
Network generation/Docker engine	Docker-compose v1.25.4/v20.10.8
GoQuorum/Tessera version	v20.10.0/v21.1.1
Client version	linux-amd64/go1.15.5
Nodejs/Npm/Python/Flask	v10.19.0/v6.14.4/v3.9.5/v2.0.1
Consensus protocol	IBFT, RAFT
Cakeshop	v0.11.0
MongoDB server	Community edition v5.0.2

**Table 5:** Deployed quorum blockchain network containers.

Type	Docker Container	Number
Quorum blockchain network core nodes	quorum node	6
	txmanager	6
	ethlogger	6
Management, monitoring, and reporting tools	cakeshop	1
	quorum reporting	1
	splunk app for quorum	1
	elasticsearch engine	1
	cadvisor	1

**Table 6:** Computational complexity of the core transactions interacting with the blockchain (read operation: *RO*, write operation: *WO*).

Transaction Type	*RO*	*WO*
E-portfolio registration	*O*(1)	*O*(1)
E-certificate issuance	*O*(2)	*O*(2)
E-certificate verification	*O*(1)	*O*(1)

**Table 7:** E-portfolio registration transaction performance.

Parameter	Value
E-portfolio registration transaction latency (ms)	164.677
Transaction size (KB)	2.380
Block size (KB)	4.251
Number of transactions per block	1
Block propagation latency (ms)	1.081

**Table 8:** E-portfolio certificate issuance and verification performance.

Parameter	Value
Ed25519 signing time per certificate (ms)	2.470
Certificate issuance transaction latency (ms)	446.744
Certificate generation time (sec)	287.931
Ed25519 verification time per certificate (ms)	139.071
Certificate file size (KB)	720
Transaction size (KB)	2.896
Block size (KB)	4.768
Number of transactions per block	1
Block propagation latency (ms)	1.705

## Data Availability

Not applicable.
